# Constructing non-stationary Dynamic Bayesian Networks with a flexible lag choosing mechanism

**DOI:** 10.1186/1471-2105-11-S6-S27

**Published:** 2010-10-07

**Authors:** Yi Jia, Jun Huan

**Affiliations:** 1 Department of Electrical Engineering & Computer Science, University of Kansas, Lawrence, KS, 66045, USA

## Abstract

**Background:**

Dynamic Bayesian Networks (DBNs) are widely used in regulatory network structure inference with gene expression data. Current methods assumed that the underlying stochastic processes that generate the gene expression data are stationary. The assumption is not realistic in certain applications where the intrinsic regulatory networks are subject to changes for adapting to internal or external stimuli.

**Results:**

In this paper we investigate a novel non-stationary DBNs method with a potential regulator detection technique and a flexible lag choosing mechanism. We apply the approach for the gene regulatory network inference on three non-stationary time series data. For the Macrophages and Arabidopsis data sets with the reference networks, our method shows better network structure prediction accuracy. For the Drosophila data set, our approach converges faster and shows a better prediction accuracy on transition times. In addition, our reconstructed regulatory networks on the Drosophila data not only share a lot of similarities with the predictions of the work of other researchers but also provide many new structural information for further investigation.

**Conclusions:**

Compared with recent proposed non-stationary DBNs methods, our approach has better structure prediction accuracy By detecting potential regulators, our method reduces the size of the search space, hence may speed up the convergence of MCMC sampling.

## Introduction

Recently non-stationary Bayesian network models have attracted significant research interests in modeling gene expression data. In non-stationary Bayesian networks, we assume that the underlying stochastic process that generates the gene expression data may change over time. Non-stationary Bayesian networks have advantage over conventional methods in applications where the intrinsic regulatory networks are subject to changes for adapting to internal or external stimuli. For example, gene expression profiles may go through dramatic changes in different development stages [1], or in the invasion process of viruses [2], or as response to changes of outside environment such as temperature and light intensity [3]. 

Recent work on non-stationary Bayesian networks could be found in [1,2]. Robinson's method [1] used RJMCMC (Reversible Jump Markov Chain Monte Carlo) to sample underlying changing network structures, in which an extended BDe metric (Bayesian-Drichlet equivalent) is applied. And Grzegorczy et al. [2] proposed a non-homogeneous Bayesian network method to model non-stationary gene regulatory processes, in which they included a Gaussian mixture model based on allocation sampler technique [4], provided an extended non-linear BGe (Bayesian Gaussian likelihood equivalent) metric and employed MCMC (Markov Chain Monte Carlo) to collect samples.

There are several limitations on the existing non-stationary DBNs methods that are discussed above. First, the RJMCMC that is used in Robinson’s work [1] is a computationally expensive approach especially in dealing with gene networks. Second, mixture model used by Grzegorczy et al. avoided intensive computational issue by using MCMC, but it does not capture the underlying changing network structures over time. In addition, both methods used a fixed time delay *τ* = 1 that leads to a relatively low accuracy of prediction on network re-construction [5].

In this paper, we proposed a new non-stationary DBNs approach extending the work presented in [1] and [5]. Our method modified RJMCMC by employing a systematic approach to determine potential regulators. We designed a flexible lag determine mechanism by considering the delay in the gene expression changes between potential regulators and target genes. In this approach we efficiently reduce the model searching space, capture the dynamics of transcriptional time delay, and speed up computation with a fast convergence.

## Related work

With a well-defined probabilistic semantics and the capability to handle hidden variables [6], Dynamic Bayesian Networks (DBNs) are widely used on regulatory network structure inference from noisy microarray gene expression data [7-16].

The early work of applying BNs to analyzing expression data could be found in [7,8]. Many works have been done since then. Hartemink et al. extended the static BNs by including latent variables and annotated edges, and their work focused on scoring the models of regulatory network [10]. Considering the problem of information loss incurred by discretization of expression data, Imoto et al. proposed a continuous BNs and non-parametric regression model [12]. They used Laplace approximation to the marginal probability to infer a BNRC score as the scoring metric for network models. Further, Hartemink and Imoto extended their techniques to DBNs [11,14]. Before the BNs, previous efforts at modeling genetic regulatory networks fell into two categories [9,10]: fine-scale methods utilizing differential equations, and coarse-scale methods using clustering and boolean network models. BNs method is perceived as a good compromise of the two levels. With the challenging of small number of samples, researchers seek additional information such as transcriptional localization data [16], DNA sequences of promoter elements [13], and protein-protein interaction data [15] to improve the accuracy of gene networks reconstruction.

## Method

### Structure Learning of Non-stationary Bayesian Networks

Bayesian networks (BNs) are a special case of probabilistic graphic models. A static BN is defined by an acyclic directed graph* G* and a complete joint probability distribution of its nodes* P*(*X*) =* P*(*X*_1_,*…, X_n_*)*.* The graph* G* :* G* = {*X, E*} contains a set of variables* X* = {*X*_1_*,…, X_n_*}, and a set of directed edges* E*, defining the causal relations between variables. With a directed acyclic graph, the joint distribution of random variables* X* = {*X*_1_,…,* X_n_*} are decomposed as* P*(*X*_1_,*…, X_n_*) = ∏*_i_ P*(*X_i_*|Π*_i_*), where* Π_i_* are the parents of the node (variable)* X_i_.*

The topology of bayesian networks must be a directed acyclic graph and hence could not be used to model the case where two genes may be a regulator of each other. As an extension of BNs to model time series data, Dynamic Bayesian Networks (DBNs) lift the limitation of directed acyclic graph by incorporating time in constructing bayesian networks. Given an observed time series data* D* spanning* T* time points, the structure learning problem of DBNs is equal to maximizing the posterior probability of the network structure* G.* By the Bayes’ rule, the posterior probability is expressed as the following:

	(1)

The current application of DBNs to gene expression data assumes that the underlying stochastic process generating the data is stationary. Here we provide a new approach to capture the structural dynamics of non-stationary data.

We assume the time series gene expression profile is subdivided to* m* segments. In each segment, there is one graph* G_i_* : 1 ≤* i *≤* m* that dominates the segment. Given a sequence of network structures *G^T^* = (*G*_1_*,…, G_m_*), the posterior probability in Equation 1 is replaced by Equation 2.

	(2)

In applying DBNs to gene expression data, we first decide the time lag value *τ*, which is the time delay between causes and effects in the time series data. Most previous work set *τ* = 1 for modeling a first-order markov chain. However, evidence shows that higher-order markov chain might better model gene expression data and biological networks [5]. Given a maximum lag value* τ_max_,* in corresponding to the graph structure sequence* G^T^*, we assign a lag vector *τ^T^* = (*τ*_1_,*…,τ_m_*), in which *τ_i_* : 1 ≤* τ_i_ ≤ τ_max_.* So Equation 2 further extends to:

	 (3)

*P*(*D\T*) is treated as a constant, and then

	(4)

In the following discussion, we specify the formula for calculating each component of Equation 4. The prior *P*(*τ_max_|T*) is 1 because we set the* τ_max_* value when we find the potential parents for each variable.

We are using the same assumption in [1] that the networks change smoothly over time. We use the exponential priors on the change of network structures. We transform the form of the sequence of graph structures *G^T^* : *G^T^* = (*G*_1_,…,*G_m_*) into *G^T^* : *G^T^* = (*G*_1_, Δ*G*_1_,…, Δ*G_m_*_−1_), where Δ*G_i_* : 1 ≤ *i* ≤ *m* −1 is the change of edges between* G_i_* and *G_i_*_+1_. we calculate* P*(*G^T^*|*m,T*) as follows.

	 (5)

,where  and* s_i_,* is the number of edges’ change between *G_i_*_+1_ and* G_i_*. We have no prior knowledge on* P*(*G*_1_) and see the uniform distribution as the prior.

We set the exponential prior on the transition times of networks over time and calculate* P*(*m|T*) as the following.

	(6)

We assume that the segments are independent and calculate* P*(*D_h_\G^T^, m, τ^T^, τ_max_,T*) of each segment as the following.

	(7)

*I_h_* is a segment where a network structure* G_h_* and its corresponding lag value* τ_h_* work.  are the parameters associated with the data of one segment* I_h_* corresponding to *G_h_*.  is the probability density function of .

We assume that the data are complete and multinomially distributed with a Dirichlet prior on the parameters. We weight the hyperparameters of Dirichlet distribution in each segment with the ratio of the segment length over the sample size. We calculate the BDe [17] score of each segment as the following:

	 (8)

*N* is the sample size of the observed data.* |I_h_|* is the length of the segment *I_h_*.  are the multinomial parameters of the joint probability distributions corresponding to *G_h_*.* r_i_* is the number of possible discrete values of* x_i_. q_ih_* is the number of configurations of parents* Π_i_* for the variable* x_i_* in the segment* I_h_. N_ijk_* (*I_h_*) is the times that* x_i_* had value* k* in the segment *I_h_*. .*α_ijk_*(*I_h_*) and* α_ij_*(*I_h_*) are the hyperparameters for Dirichlet distributions applied in the segment *I_h_*. *α_ijk_*(*I_h_*) is assumed to be uniformly distributed inside a segment and is set to *α_ijk_*(*I_h_*) = *α*|*I_h_*|/(*r_i_q_ih_N*) *α* is the equivalent sample size. We calculate the marginal likelihood *P*(*D*|*G^T^*, *m, τ^T^*, *τ_max_*, *T*) by using the modified Bayesian-Dirichlet equivalent ( BDe ) metric introduced in [1]. By multiplying the BDe metric of each segment, we get the extended BDe metric equation as follows:

	(9)

Once the parents are decided, we use a conditional probability vector  with 	 So P(*τ^T^* |m, *τ_max_*, *T*) is calculated by:

	 (10)

where  is the conditional probability of the* j*th component’s value in the lag vector* τ^T^*.

### Potential regulator detection

**Figure 1:**
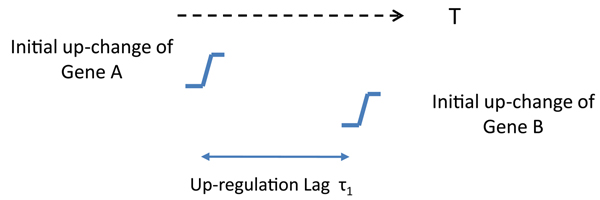
One example of detecting a potential up-regulation pair *A → B*.

We know that the change of expression level of most transcriptional factors (TFs) always precedes or happens simultaneously with that of target genes [18]. This fact provides a useful technique to find potential regulators and relative expression lag value* τ*. We follow Zou’s work [5] to detect the possible TFs. In Zou’s work, they used the expression levels of ≥ 1.2-fold and ≤ 0.70-fold compared with the average gene expression level as up-regulation and down-regulation cutoff thresholds. Any gene with initial up(down) change of expression level earlier is seen as the potential TFs of genes with change of expression level later. One example of up-regulation is showed in Figure 1. Instead of using a fixed value we relax the cutoff thresholds by taking a range of values. For up-regulation, we use the range 1.0 ~ 1.2, and for down-regulation, we take the range 0.6 ~ 0.8. In order to get all the possible TFs for each gene, we need to consider all the combinations of possible up(down)-regulation pairs. The yeast cell cycle data set analyzed by Zou has a limited time points (*T* = 16), which makes the complete search over all possible lag values affordable. However, with the increasing sample size and number of genes in the gene expression profiles, this searching algorithm is unrealistic and will bring more noises and high computational cost. We developed a heuristic to limit the potential regulator-target gene pairs for processing large data sets.

**Figure 2:**
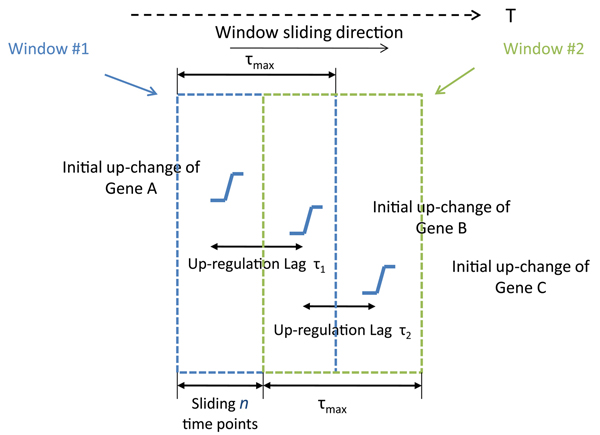
**One example of the sliding window.** With window 1, we found the potential up-regulation pair *A → B*. After sliding *n* time steps, with window 2, we identified *B → C*.

Below is our method. We first discretize the expression data by following the method above. We then search the data and only select the initial up(down)-regulation points. Slide the window with the width *τ_max_* from the start (*t* = 1) of the time series expression data to the end (*t* = T − *τ_max_* + 1), where* T* is the length of time points. For each moving step, the window slides one time step and only the up(down)-regulation pairs inside the window are calculated. One example of the sliding window is showed in Figure 2. We group the pairs according to their time lag and calculate the posterior probability for each lag value* τ* : 1 ≤* τ ≤ τ_max_.* For each gene, its potential TFs are also collected to be used as the prior knowledge to limit the search space during the process of structure sampling.

### Structure sampling using RJMCMC

We choose sampling approaches rather than heuristic methods to search network structures due to the reason that microarray expression data are usually sparse, which makes the posterior probability of structures to be diffuse [9]. In this approach, a group of most likely structures could explain data better than a single one. We use a sampling method called RJMCMC (Reversible Jump Markov Chain Monte Carlo) to collect structure samples. The details of this method are available on [19].

Compared with the move types introduced in [1], we add one new move type called* change lag* and modify most of the existing operations by incorporating more restrictions. We also define a vector of time points *L^T^ =* (*L*_1_*,...,L_m_*_−1_), where* L_i_* : 1 ≤* i ≤ m −* 1 is the start time point where* G_i+_*_1_ is applied. We use Metropolis-Hastings algorithm for RJMCMC sampling [20]. The move set of our RJMCMC consists of 11 move types:

MT1:* add edge to G_i_.*

MT2:* delete edge from G_i_.*

MT3:* add edge to* Δ*G_i_.*

MT4:* delete edge from* Δ*G_i_.*

MT5:* move edge between* Δ*G_i_*s.

MT6:* shift time,* which changes a single *L_i_*’s value. This operation will trigger the checking of *τ_i_*’s value under the restriction of* τ_i_ ≤ L_i_ −* 2, where 1 ≤* i ≤ m −* 1, and* τ_m_ ≤ T −* 1.

MT7:* change lag,* which changes a single* τ_i_*’s value. This move type needs to follow the limitations showed on MT6.

MT8:* merge* Δ*G_i_ and* Δ*G_i+_*_1_.

MT9:* split* Δ*G_i_*.

MT10:* create new* Δ*G_i_*.

MT11:* delete* Δ*G_i_.*

Both MT8 and MT9 operations will trigger the change of dimensions of* L^T^* and *τ^T^*. In MT8, the new component of* τ^T^* takes the least value of two merged components. Similarly with MT8 and MT9, M10 and M11 will change the dimensions of* L^T^* and* τ^T^*. MT1, MT3, MT10 and MT11 follow the restriction that the edges pointed to one target gene should have the origins from its potential regulators.

## Experimental study and evaluation

We performed all the experiments on a cluster with 256 Intel Xeon 3.2 Ghz EM64T processors with 4 GB memory each. We implemented our method FLnsDBNs (Flexible Lag Non-Stationary Dynamic Bayesian Networks) in Matlab.

We compare three approaches: our approach FLnsDBNs, reversible jump Markov chain Monte Carlo Non-Stationary Dynamic Bayesian Networks (RJnsDBNs) [1], and Allocation Sampler Non-Stationary Dynamic Bayesian Networks (ASnsDBNs) [2]. For RJnsDBNs, we use the default setting of unknown numbers and times of transitions (UNUT) in all of the data sets. RJnsDBNs is implemented in Java, and ASnsDBNs is implemented in Matlab. We show the average elapsed time of three methods on two data sets in Table 1. In FLnsDBNs, we ignore the computational cost on the potential regulator detection process because it takes less than 0.03 second. Although the direct comparison of three approaches by using the elapsed time is unfair due to the difference in implementation, our method shows the comparable computational performance with ASnsDBNs.

**Table 1:** The computational time of three methods

	*CMV*	*ArobidopsisThalianaT 20*
RJnsDBNs	9.06s	333s
ASnsDBNs	457.53s	13394s
FLnsDBNs	219.66s	14034s

The parameter configurations of three methods are shown in Figure 4 and 7.

Our experimental study is based on three data sets: (i) Bone Marrow-derived Macrophages gene expression time series data (Macrophages data set), (ii) Circadian regulation in Arabidopsis Thaliana gene expression time series data (Arabidopsis data set), and (iii) Drosophila muscle development gene expression time series data (Drosophila data set). To compare the results from different data sets, we follow the evaluation method introduced in [2,9,21]. For each data set, we first collect gold standard reference networks as the ground truth. For the Macrophages data set, such reference networks are available in [2,22,23]. For the Arabidopsis data set, we collect the network information from [3,24-27]. For the Drosophila data set, there is no ground truth regarding the network structure. We compare our method with others by showing the commonality and differences. In case where we have ground truth network structure (the Bone Marrow data set and Arabidopsis data set), we use the area under receiver operating characteristic curve (AUROC) values to evaluate the performance. We obtained the ROC curves by postprocessing the posterior probabilities of directed edges and taking different cutoff thresholds in [0, 1]. If the posterior probability of an edge is greater than the threshold, we keep the edge. Otherwise, we do not keep the edge. With the ROC curves, we evaluate the performance of different methods by comparing the AUROC scores. In addition, for each data set, we show the posterior distribution of the number of segments and the locations of changepoints. In all of our experimental study, we find that the method FLnsDBNs produces compatible results with previous methods and demonstrates better network prediction performance in all the data sets. Before we discuss the details of experimental results, we present our data set first below.

### Data sets

As mentioned briefly before, we evaluate our method on three data sets used in [1,2]. We preprocess the original data sets by following Zhao’s work [28]. We set the values of a missed time point with the mean of its two neighbors; i.e.,* X_i_*,*_t_* = (*X_i_*,*_t_*_−1_ + *X_i_*_,_*_t_*_+1_)/2 if 1* < t < T*. If the missed values are at the beginning or end, simply set the same value as its neighbor; i.e.,* X_i_*_,_*_t_* =* X_i_*_,_*_t_*_+1_ if* t* = 1 or* X_i_*_,_*_t_* =* X_i_*_,_*_t_*_−1_ if* t* =* T*. In the following, we show the details of each data set.

**Bone Marrow-derived Macrophages gene expression data.** Interferon regulatory factors (IRFs) are proteins crucial for the mammalian innate immunity [29]. These transcription factors are central to the innate immune response to the infection by pathogenic organisms [23]. We use the Macrophage data sets sampled from three external conditions: (I) Infection with Cytomegalovirus (CMV), (II) Treatment with Interferon Gamma (*IFN_γ_*), and (III) Infection with Cytomegalovirus after pretreatment with* IFN_γ_* (C*MV+IFN_γ_*). Each data set has 3 genes:* Irf1, Irf2* and* Irf3,* and contains 25 time points with the interval of 30 minutes. We use the network* Irf* 2* ↔ Irf* 1* ← Irf* 3 as the gold standard and assume the network never changes over the time.

**Figure 3:**
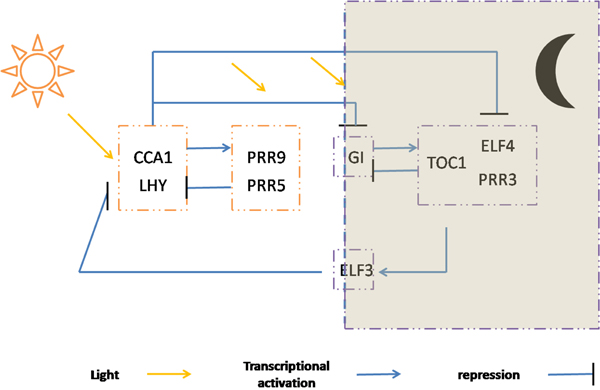
The A. thaliana oscillator loops of the circadian clock network.

**Arabidopsis thaliana circadian regulation gene expression data.** A. thaliana circadian gene expression data was sampled to understand the internal clock-signalling network of plant. Two data sets were collected with the interval of 2h from two light-dark conditions: 10h:10h and 14h:14h light/dark cycles, both of which contain 13 time points. We choose a group of 9 genes,* LHY, CCA1, TOC1, ELF4, ELF3, GI, PRR9, PRR5,* and* PRR3* for analysis, which create transcriptional feedback loops. We show the referred biological regulatory network in Figure 3. In this network,* CCA1, LHY* and* TOC1,* as core components of the reciprocal regulation , are important for the proper function of this oscillator network in A. thaliana [3].* CCA1* and* LHY* proteins’ direct binding to the promoter of* TOC1* represses the expression of* TOC1,* and* ELF3* works as a negative regulator of light signaling to the clock oscillator and enables the induction of oscillator output [24,25]. The pseudo-response regulators PRR5 and PRR9 are activated by* CCA1* and* LHY* accompanied with light, and repress* CCA1* and* LHY* subsequently.* G1* is activated by light and improve the expression of* TOC1. ELF4* is repressed by* CCA1.* And* PRR3* is highly correlated with* TOC1* and together form a functional complex [30].

**Drosophila muscle development gene expression data.** The original transcriptional profile on the life cycle of Drosophila melanogaster contains 4028 genes, nearly one third of all of the predicted Drosophila genes. The samples were collected over 66 time steps throughout the life cycle of Drosophila melanogaster consisting of four periods: embryonic, larval, pupal, and adulthood periods [31]. The intervals of sampling are not even, from overlapped 1 hour during the early embryonic period to multiple days in the adulthood. We choose 11 genes for analysis, which are* eve, gfl/lmd, twi, mlc1, sls, mhc, prm, actn, up, myo61f, msp300.* Those genes were reported to be related with the muscle development of Drosophila.

### Experimental results

In this section, we compare the experimental results of three approaches: FLnsDBNs, RJnsDBNs, and ASnsDBNs on three data sets.

**Figure 4:**
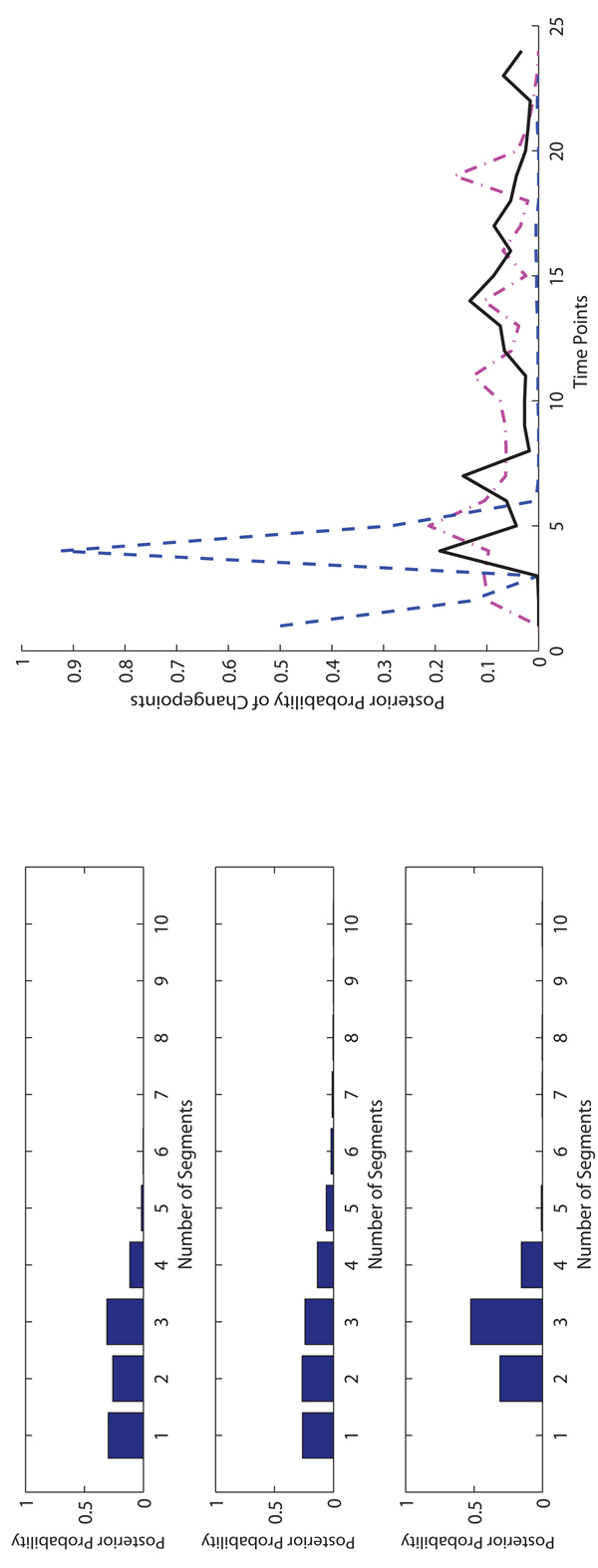
**Comparison of three methods on* CMV* Macrophage data.** Left: The posterior probabilities of the numbers of segments (top: FLnsDBNs (*λ_m_* = 4.05, λ*_s_* = 2); middle: RJnsDBNs (*λ_m_* = 0.65, λ*_s_* = 2); bottom: ASnsDBNs). Right: The posterior probabilities of the change points (FLnsDBNs: black solid line; RnsDBNs: magenta dash-dot line; ASnsDBNs: blue dashed line).

**Figure 5:**
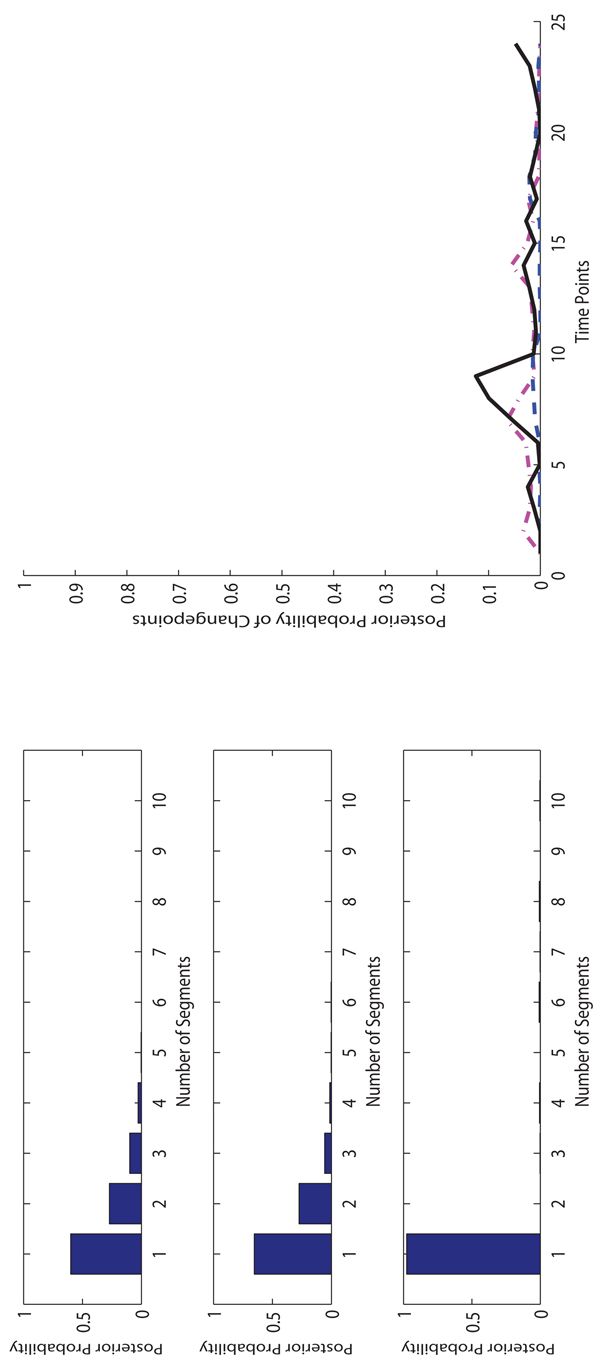
**Comparison of three methods on* CMV* +* IFN_γ_* Macrophage data.** Left: The posterior probabilities of the numbers of segments (top: FLnsDBNs* (λ_m_ =* 6,* λ_s_* = 2); middle: RJnsDBNs* (λ_m_* = 1, λ_s_ = 2); bottom: ASnsDBNs). Right: The posterior probabilities of the change points (FLnsDBNs: black solid line; RnsDBNs: magenta dash-dot line; ASnsDBNs: blue dashed line).

**Figure 6:**
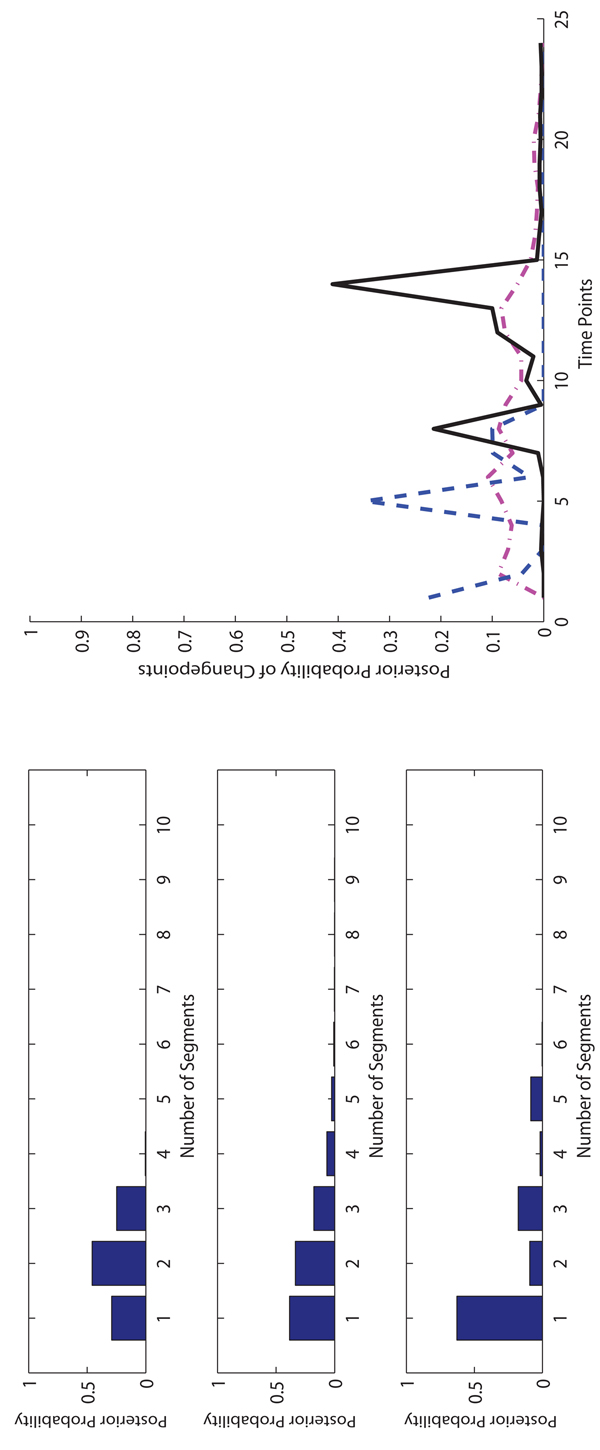
**Comparison of three methods on* IFN_γ_* Macrophage data.** Left: The posterior probabilities of the numbers of segments (top: FLnsDBNs (*λ_m_* = 6.5, λ*_s_* = 2); middle: RJnsDBNs (λ*_m_* = 0.001, λ*_s_* = 2); bottom: ASnsDBNs). Right: The posterior probabilities of the change points (FLnsDBNs: black solid line; RnsDBNs: magenta dash-dot line; ASnsDBNs: blue dashed line).

**The experimental results on Macrophages data.** On the Macrophages data, for each method, we run 10,000 iterations for burn-in and then take additional 40,000 iterations to collect samples. In Figure 4, 5 and 6, we show the posterior probabilities of the numbers of segments and changepoints on three Macrophages data sets. The sample collection of FLnsDBNs on the Macrophages data takes about 2 minutes.

For the* CM V* data, we first observe that there is a high agreement among all three methods in term of the range of the number of identified segments. The ranges are 1 ~ 4 for FLnsDBNs, 1 ~ 4 for RJnsDBNs, and 2 ~ 4 for ASnsDBNs. When we compare the distributions of the number of segments identified by three methods, we observe that ASnsDBNs clearly identifies a dominant 3-segment in the data set while the posterior probabilities produced by FLnsDBNs and RJnsDBNs are flat. For the predicted locations of the changepoints, FLnsDBNs identifies three posterior peaks at time stamps 4, 8, and 14. RJnsDBNs finds four peaks at 5, 11, 14, and 19. In ASnsDBNs, two peaks happen at 1 and 4 with the probabilities more than 0.5. There is a consensus among three methods that the most probable changepoint occurs at the location 4. The results of three methods are consistent with the biological phenomenon that the simultaneous responses of Macrophages happen under the attack of Cytomegalovirus [2]. In order to assess the network prediction performance, we show the AUROC scores in Table 2. We find that all methods perform well in the CMV data with the AUROC scores equal to 1.

**Table 2:** Comparison of AUROC values on Macrophage data

	*CMV*	*IFN_γ_*	*CMV + IFN_γ_*
RJnsDBNs	1	0.7778	0.2222
ASnsDBNs	1	0.6667	0.6667
FLnsDBNs	1	0.8333	1

TP, true positive; FP, false positive; TN, true negative; FN, false negative.

Sensitivity = TP/(TP+FN).

Specificity = TN/(TN+FP)

Complementary Specificity = 1- Specificity = FP/(TN+FP). The ROC curves are plotted with the Sensitivity scores against the corresponding Complementary Specificity scores.

For the* CMV* +* IFN_γ_* data, all three methods identify 1 segment, which corresponds to a coexistence state between virus and its host cell [2,32], and have the same range of the number of segments 1 ~ 3. In Table 2, we find that FLnsDBNs shows a much better network prediction with the AUROC score equal to 1 while in RJnsDBNs the AUROC score is equal to 0.2222 and in ASnsDBNs the AUROc score is equal to 0.6667. For the* IFN_γ_* data, there is a postulated transition with the immune activation under the treatment of *IFNγ*. FLnsDBNs infers 2 segments and finds two posterior peaks of transition time at 8 and 14. 

ASnsDBNs and RJnsDBNs infer only one segment, even though the two methods identify a differnt posterior peak at the location around 5. On the assessment of the predicted network structures, the AUROC scores are 0.8333 in FLnsDBNs, 0.7778 in RJnsDBNs, and 0.6667 in ASnsDBNs. In all of three Macrophages data sets, our approach shows the best network prediction accuracy.

For each Macrophages data set using FLnsDBNs and RJnsDBNs methods, we find that the posterior probability distributions of any edge do not change much across different segments. This finding is consistent with the assumption that the underlying network does not change through the time.

**The experimental results on Arabidopsis data.** On the Arabidopsis data, we use a larger number of iterations in the MCMC sampling because the data set is much larger than the Macrophages data. We run 10,000 iterations for burn-in and then take additional 990,000 iterations to collect samples. The sample collection of FLnsDBNs on the Arabidopsis data takes about 4 hours.

**Figure 7:**
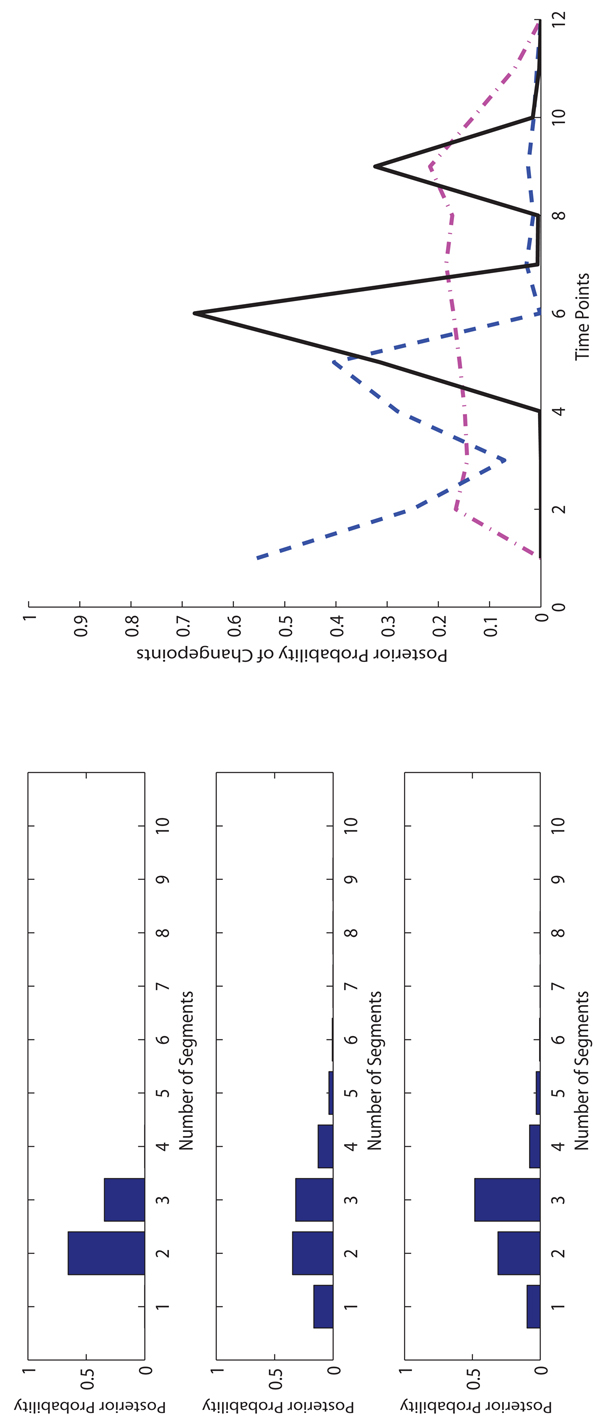
**Comparison of three methods on Arabidopsis T20 data.** Left: The posterior probabilities of the numbers of segments (top: FLnsDBNs (λ*_m_* = 14, λ*_s_* = 2); middle: RJnsDBNs* (λ_m_* = 0.0005, λ*_s_* = 2); bottom: ASnsDBNs). Right: The posterior probabilities of the change points (FLnsDBNs: black solid line; RnsDBNs: magenta dash-dot line; ASnsDBNs: blue dashed line).

**Figure 8:**
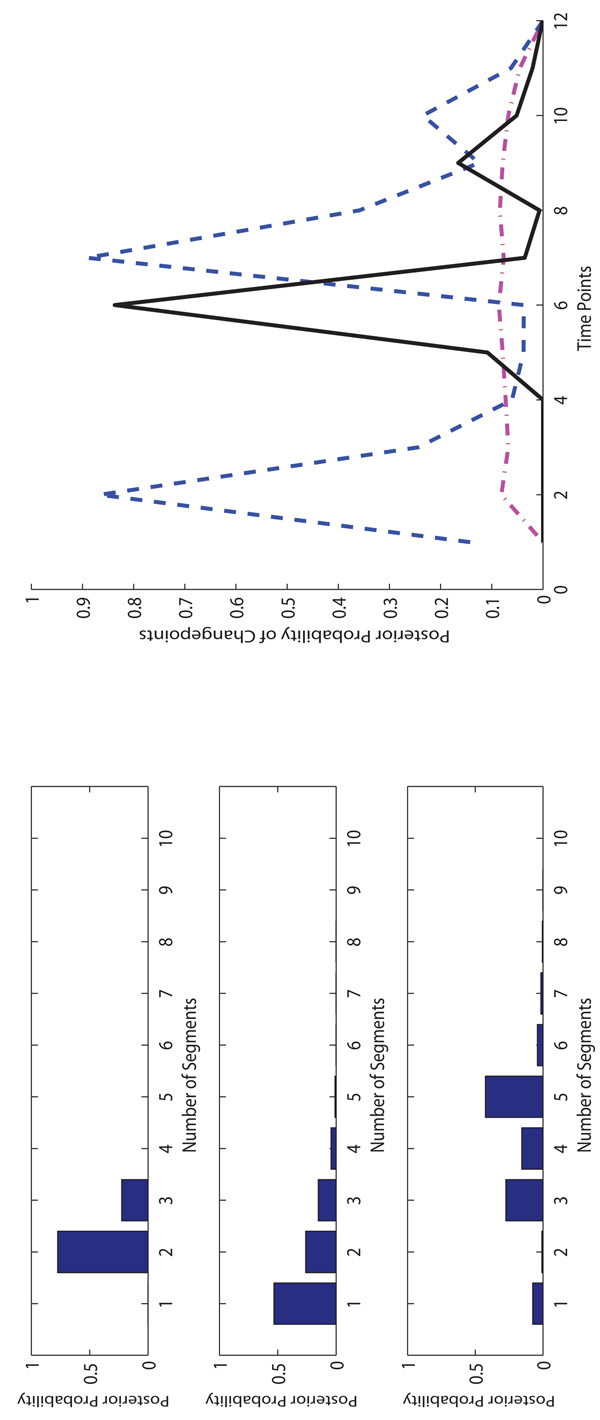
**Comparison of three methods on Arabidopsis T28 data.** Left: The posterior probabilities of the numbers of segments (top: FLnsDBNs (λ*_m_* = 14, λ*_s_* = 2); middle: RJnsDBNs* (λ_m_* = 0.005, λ*_s_* = 2); bottom: ASnsDBNs). Right: The posterior probabilities of the change points (FLnsDBNs: black solid line; RnsDBNs: magenta dash-dot line; ASnsDBNs: blue dashed line).

In Figure 7 and 8, we show the posterior distributions of the numbers of segments and changepoints on two Arabidopsis data sets. For the Arabidopsis T20 data, in FLnsDBNs the range of the number of segments is 2 ~ 3, and in RJnsDBNs and ALnsDBNs the ranges are 1 ~ 4. In FLnsDBNs, the dominant samples are the ones with 2 segments while in AlnsDBNs they are 3 segments. For the Arabidopsis T28 data, the ranges are 2 ~ 3 in FLnsDBNs, 1 ~ 3 in RJnsDBNs and 3 ~ 5 in ASnsDBNs. FLnsDBNs infers 2 segments, RJnsDBNs infers 1 segment, and ASnsDBNs infers 5 segments, respectively on the T28 data. In both data sets, we find that the differences of the posterior probabilities of 2 and 3 segments are low in RJnsDBNs and the difference between the posterior peaks of changepoints and the time points nearby are not noticeable. Hence, for this data set, we only use a single network in RJnsDBNs to compare with other methods. Using ASnsDBNs, the poseterior peaks of changepoints on T20 data are 1, 5 and those on T28 are 2, 7, 10. In [2], the results of ASnsDBNs are explained as a phase shift incurred by different dark/light cycles. However, our approach predicts the posterior peak of changepoints both at the location 6. We evaluated the network reconstruction accuracy of three methods by comparing with the reference network showed in Section 3.2. We show the AUROC scores in Table 3. In addition, we use a new comparative criteria called the TP|FP=5 counts [2,21] to further demonstrate the performance of our method. TP are the true positive counts; FP are the false positive counts; TP|FP=5 are the TP counts when FP is 5. The TP|FP=5 counts of three approaches are shown in Table 4. FLnsDBNs outperforms other two methods in both two evaluation criteria of the AUROC score and TP|FP=5 counts on the Arabidopsis data sets.

**Table 3:** Comparison of AUROC values on Arabidopsis data

	*ArabidopsisT 20*	*ArabidopsisT 28*
RJnsDBNs	0.5070	0.5773
ASnsDBNs	0.5929	0.5641
FLnsDBNs	G1:0.6138; G2:0.6150	G1:0.6558; G2:0.6628

TP, true positive; FP, false positive; TN, true negative; FN, false negative.

Sensitivity = TP/(TP+FN).

Specificity = TN/(TN+FP).

Complementary Specificity = 1- Specificity = FP/(TN+FP). The ROC curves are plotted with the Sensitivity scores against the corresponding Complementary Specificity scores. G1 and G2 are two networks reconstructed based on the changepoint 6.

**Table 4:** Comparison of* TP|FP* = 5 values on Arabidopsis data

	*ArabidopsisT 20*	*ArabidopsisT 28*
RJnsDBNs	2	6
ASnsDBNs	4	3
FLnsDBNs	G1:8; G2:8	G1:11; G2:11

G1 and G2 are two reconstructed networks separated by the changepoint 6.

**The experimental results on Drosophila data.** For the Drosophila data, We run 10,000 iterations for burn-in and then take additional 990,000 iterations to collect samples. The sample collection of FLnsDBNs on the Drosophila data takes about 10 hours.

**Figure 9:**
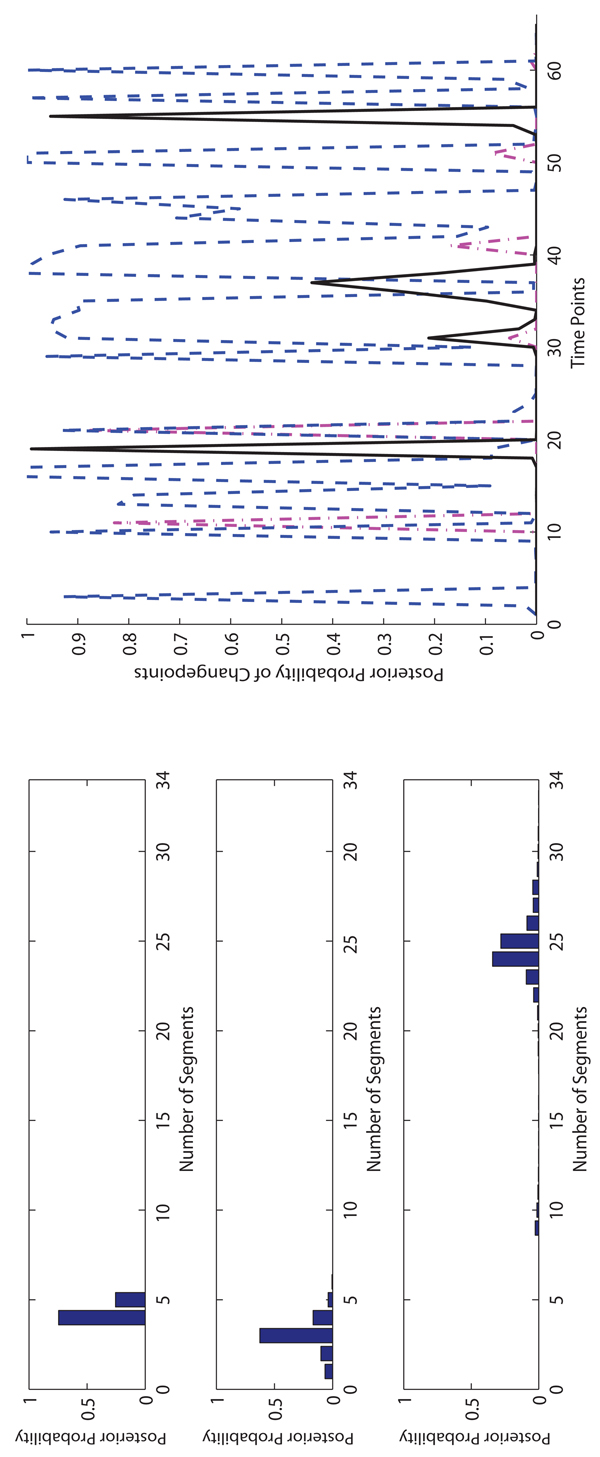
**Comparison of three methods on drosophila data.** Left: The posterior probabilities of the numbers of segments (top: FLnsDBNs (λ*_m_* = 35, λ*_s_* = 2); middle: RJnsDBNs* (λ_m_ =* 2,* λ_s_* = 2); bottom: ASnsDBNs). Right: The posterior probabilities of the change points (FLnsDBNs: black solid line; RnsDBNs: magenta dash-dot line; ASnsDBNs: blue dashed line).

We show the results of posterior probabilities of the numbers of segments and changepoints in Figure 9. ASnsDBNs predicts more than 20 segments and fails to provide a meaningful result of changepoints. Therefore, in the subsequent discussion, we only compare FLnsDBNs and RJnsDBNs approaches. The assumed transition time of four life periods are located at 30, 40 and 58. RJnsDBNs predicts 3 segments with the posterior peaks located at 11 and 21. FLnsDBNs prefers 4 segments with the posterior peaks at 19, 36 and 54, which happen before the assumed changepoints. And our prediction of the Embryonic→Larval transition occurs at 19 much earlier than 30. Both ASnsDBNs and RJnsDBNs methods do not converge well in this fly data set.

**Figure 10:**
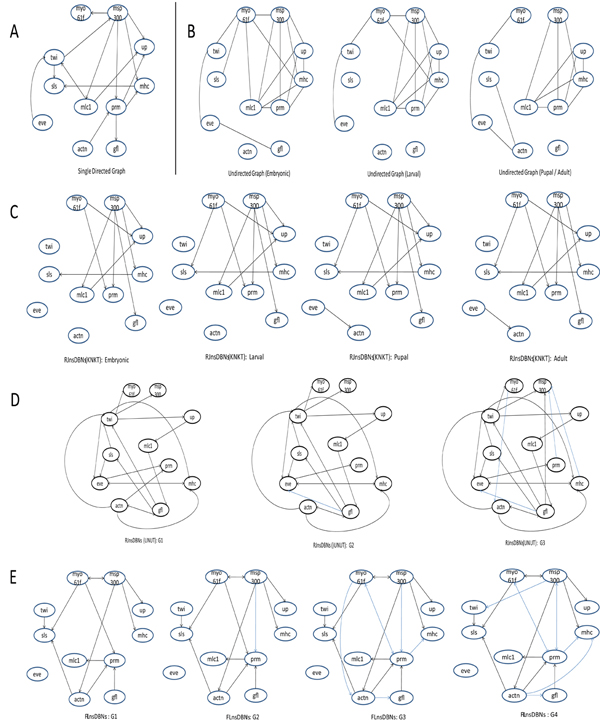
**Comparison of reconstructed networks on drosophila data.** A: The stationary directed networks predicted by [33]. B: The non-stationary undirected networks predicted by [34]. C: The non-stationary undirected networks predicted by RJnsDBNs with the setting of KNKT (λ*_s_* = 2) [1]. The posterior probability cutoff threshold 0.5 is used. D: The non-stationary undirected networks predicted by RJnsDBNs with the setting of UNUT (λ*_m_* = 2, λ*_s_* = 2). The posterior probability cutoff threshold 0.5 is used. The edges in blue color is the new edges different from G1. E: The non-stationary undirected networks predicted by FLnsDBNs (*λ_m_* = 35, λ*_s_* = 2). The posterior probability cutoff threshold 0.3 is used. The edges in blue color is the new edges different from the first graph G1.

We show the reconstructed networks of our approach, those of RJnsDBNs (UNUT), a stationary directed network predicted by [33], and the non-stationary undirected networks predicted by [34] in Figure 10 for the purpose of comparison. In addition, we provide the networks predicted by RJnsDBNs with another setting of KNKT to compare because the networks inferred by RJnsDBNs (UNUT) show much difference from other predictions. In the following, we only compare the results of [34], [33], RJnsDBNs (KNKT) and FLnsDBNs.

These four predictions share many similarities and also show some difference. We find that the gene *msp-300* may play a key role in the cluster of these 11 genes.* myo-61f* is only predicted to be a regulated gene by* msp-300* in [33], but other three methods show that* myo-61f* is another key gene in this cluster. In [33],* myo-61f* is correlated with* twi, sls, mlc1, mhc* and* msp-300.* In RJnsDBNs (KNKT),* myo-61f* serves as the regulators of *prm*,* up* and *sls*. Our approach predicts that* myo-61f* regulates four genes:* sls, prm, actn,* and* msp-300.* FLnsDBNs, [33] and [34] all agree that there are regulation relationships between *myo-61f* and* msp300,* while RJnsDBNs (KNKT) did not identify this interaction. Different from the prediction of RJnsDBNs (KNKT), Our approach finds that* twi* is not separated from other genes and* actn* serves as the parents of other genes, which is consistent with the networks in [33]. In Figure 10E,* twi* is the regulator of* sls,* and* actn* regulates* sls, prm* and* gfl.* We also notice that the regulating effects of* myo-61f* and* msp-300* on other genes intensify over the time. Nearly different from all of three methods, our approach finds that* twi* and* gfl/lmd* are regulators of other genes while only [33] sees* twi* as a regulator. *gfl/lmd* and* twi* are direct upstream regulators of* mef2*[35,36] that directly regulates some target myosin family genes at all stages of muscle development [37], such as* mhc* and* mlc1.* Evidence show the cooperative binding of* twi* and* Mef2* or* gfl/lmd* and* Mef2* to these target genes are attractive models [35,37]. It indicates that a co-regulation role of* twi* and* gfl/lmd* with* Mef2* to other muscle development genes may exist. The prediction of our method shows this biological behavior. Currently the reference regulatory network on the muscle development of Drosophila melanogaster is not available and the relevant biological literatures are limited. Further biological researches and experiments are needed to verify the regulatory networks.

## Conclusion

In this paper we introduced a new non-stationary DBNs method and applied our approach on three time series microarray gene expression data. Our new DBNs method uses a systematic way to determine potential regulators and takes a flexible lag choosing mechanism. Our experimental study demonstrated that compared with recent proposed non-stationary DBNs methods, our approach has better structure prediction accuracy. By detecting potential regulators, our method reduces the size of the search space, hence may speed up the convergence of MCMC sampling.

## Competing interests

The authors declare that they have no competing interests.

## Authors’ contributions

YJ developed methods, implemented the software, and drafted the manuscript. JH was responsible for all aspects of the project, and helped revise the manuscript.

## References

[B1] Robinson JW Hartemink AJ Non-stationary dynamic Bayesian networks. Procedding of Advances in Neural Information Processing Systems Conference 2008

[B2] Grzegorczy M Husmeier D Edwards KD Ghazal P Millar AJ Modelling non-stationary gene regulatory processes with a non-homogeneous Bayesian network and the allocation sampler. Bioinformatics 2008 24 2071 2078 10.1093/bioinformatics/btn367 18664467

[B3] Mas P Circadian clock function in Arabidopsis thaliana: time beyond transcription. Trends Cell Biol 2008 18 273 181 10.1016/j.tcb.2008.03.005 18468438

[B4] Nobile A Fearnside AT Bayesian finite mixtures with an unknown number of components: The allocation sampler. Statistics and Computing 2007 17 147 162 10.1007/s11222-006-9014-7

[B5] Zou M Conzen SD A new dynamic Bayesian network (DBN) approach for identifying gene regulatory networks from time course microarray data. Bioinformatics 2004 21 71 79 10.1093/bioinformatics/bth463 15308537

[B6] McAdams HH Arkin A Stochastic mechanisms in gene expression. Proc Natl Acad Sci U S A 1997 94 3 814 819 10.1073/pnas.94.3.814 9023339 PMC19596

[B7] Friedman N Linial M Nachman I Pe’er D Using Bayesian networks to analyze expression data. Journal of Computational Biology 2000 7 3-4 601 620 10.1089/106652700750050961 11108481

[B8] Murphy K Mian S Modeling gene expression data using dynamic Bayesian networks. Technical Report 1999

[B9] Husmeier D Sensitivity and specificity of inferring genetic regulatory interactions from microarray experiments with dynamic Bayesian networks. Bioinformatics 2003 19 2271 2282 10.1093/bioinformatics/btg313 14630656

[B10] Hartemink AJ Gifford DK Jaakkola TS Young RA Using graphical models and genomic expression data to statistically validate models of genetic regulatory networks. Proceedings of Pacific Symposium on Biocomputing 2001 422 433 10.1142/9789814447362_0042 11262961

[B11] Yu J Smith VA Wang PP Hartemink AJ Jarvis ED Advances to Bayesian network inference for generating causal networks from observational biological data. Bioinformatics 2004 20 3594 3603 10.1093/bioinformatics/bth448 15284094

[B12] Imoto S Goto T Miyano S Estimation of genetic networks and functional structures between genes by using Bayesian networks and nonparametric regression. Proceedings of Pacific Symposium on Biocomputing 2002 175 186 11928473

[B13] Imoto S Higuchi T Goto T Tashiro K Kuhara S Miyano S Combining microarrays and biological knowledge for estimating gene networks via Bayesian networks. Computer Society Bioinformatics Conference (CSB’03) 2003 104 16452784

[B14] Kim SY Imoto S Miyano S Inferring gene networks from time series microarray data using dynamic Bayesian networks. Brief Bioinform 2003 4 228 235 10.1093/bib/4.3.228 14582517

[B15] Nariai N Kim SY Imoto S Miyano S Using protein-protein interactions for refining gene networks estimated from Microarray data by Bayesian networks. Pacific Symposium on Biocomputing 2004 9 336 347 10.1142/9789812704856_0032 14992515

[B16] Bernard A Hartemink AJ Informative structure priors: joint learning of dynamic regulatory networks from multiple types of data. Proceedings of Pacific Symposium on Biocomputing 2005 459 70 full_text 15759651

[B17] Heckerman D Geiger D Chickering DM Learning Bayesian networks: The combination of knowledge and statistical data. Machine Learning 1995 20 3 197 243

[B18] Yu H Luscombe NM Qian J Gerstein M Genomic analysis of gene expression relationships in transcriptional regulatory networks. Trends Genet 2003 19 422 7 10.1016/S0168-9525(03)00175-6 12902159

[B19] Green PJ Reversible jump Markov chain Monte Carlo computation and Bayesian model determination. Biometrika 1995 82 711 732 10.1093/biomet/82.4.711

[B20] Chib S Greenberg E Understanding the Metropolis Hasting Algorithm. Amer. Statist 1995 49 327 335 10.2307/2684568

[B21] Werhli AV Grzegorczyk M Husmeier D Comparative evaluation of reverse engineering gene regulatory networks with relevance networks, graphical gaussian models and bayesian networks. Bioinformatics 2006 22 20 2523 2531 10.1093/bioinformatics/btl391 16844710

[B22] JD Jr Kerr I Stark G Jak-STAT pathways and transcriptional activation in response to IFNs and other extracellular signaling proteins. Science 1994 264 1415 1421 10.1126/science.8197455 8197455

[B23] Raza S Robertson KA Lacaze PA Page D Enright AJ Ghazal P Freeman TC A logic-based diagram of signalling pathways central to macrophage activation. BMC Syst Biol 2008 2 36 10.1186/1752-0509-2-36 18433497 PMC2383880

[B24] Salome PA McClung CR The Arabidopsis thaliana Clock. Journal of Biological Rhythms 2004 19 5 425 435 10.1177/0748730404268112 15534322

[B25] Covington MF Panda S Liu XL Strayer CA Wagner DR Kay SA ELF3 Modulates Resetting of the Circadian Clock in Arabidopsis. The Plant Cell 2001 13 1305 1315 10.2307/3871297 11402162 PMC135573

[B26] Hall A Kozma-Bognar L Reka Toth Nagy F Millar AJ Conditional circadian regulation of PHYTOCHROME A gene expression. Plant Physiol 2001 127 4 1808 18 10.1104/pp.010294 11743124 PMC133584

[B27] Mizuno T Nakamichi N Pseudo-Response Regulators (PRRs) or True Oscillator Components (TOCs). Plant Cell Physiol 2005 46 5 677 685 10.1093/pcp/pci087 15767264

[B28] Zhao W Serpedin E Dougherty ER Inferring gene regulatory networks from time series data using the minimum description length principle. Bioinformatics 2006 22 17 2129 2135 10.1093/bioinformatics/btl364 16845143

[B29] Honda K Takaoka A Taniguchi T Type I Interferon Gene Induction by the Interferon Regulatory Factor Family of Transcription Factors. Immunity 2006 25 349 360 10.1016/j.immuni.2006.08.009 16979567

[B30] Para A Farre EM Imaizumi T Pruneda-Paz JL Harmon FG Kay SA PRR3 Is a vascular regulator of TOC1 stability in the Arabidopsis circadian clock. Plant Cell 2007 19 11 3462 73 10.1105/tpc.107.054775 18055606 PMC2174887

[B31] Arbeitman MN Furlong EEM Imam F Johnson E Null BH Baker BS Krasnow MA Scott MP Davis RW White KP Gene Expression During the Life Cycle of Drosophila melanogaster. Science 2002 297 5590 2270 2275 10.1126/science.1072152 12351791

[B32] Benedict CA Banks TA Senderowicz L Ko M Britt WJ Angulo A Ghazal P Ware CF Lymphotoxins and Cytomegalovirus cooperatively Induce Interferon-b Establishing Host-Virus Detente. Immunity 2001 15 617 626 10.1016/S1074-7613(01)00222-9 11672543

[B33] Zhao W Serpedin E Dougherty ER Inferring gene regulatory networks from time series data using the minimum description length principle. Bioinformatics 2006 22 17 2129 2135 10.1093/bioinformatics/btl364 16845143

[B34] Guo F Hanneke S Pu W Xing EP Recovering temporally rewiring networks: A model-based approach. ICML 2007 24

[B35] Duan H Nguyen HT Distinct Posttranscriptional Mechanisms Regulate the Activity of the Zn Finger Transcription Factor Lame duck during Drosophila Myogenesis. Mol Cell Biol 2006 26 4 1414 1423 10.1128/MCB.26.4.1414-1423.2006 16449652 PMC1367186

[B36] Cripps RM Black BL Zhao B Lien CL Schulz RA Olson EN The myogenic regulatory gene Mef2 is a direct target for transcriptional activation by Twist during Drosophila myogenesis. Genes Dev 1998 12 3 422 34 10.1101/gad.12.3.422 9450935 PMC316486

[B37] Sandmann T Jensen LJ Jakobsen JS Karzynski MM Eichenlaub MP Bork P Furlong EE DA temporal map of transcription factor activity: mef2 directly regulates target genes at all stages of muscle development. Dev Cell 2006 10 6 797 807 10.1016/j.devcel.2006.04.009 16740481

